# PB.51: Axillary lymph node ultrasound and fine needle aspiration in preoperative staging of breast cancer: re-audit

**DOI:** 10.1186/bcr3551

**Published:** 2013-11-08

**Authors:** H Kazi, H Humphreys, L Clarke, A Robinson, A Leaver

**Affiliations:** 1 South Tyneside Hospital, South Shields, UK; 2 Queen Elizabeth Hospital, Gateshead, UK

## Introduction

Axillary ultrasound and fine needle aspiration (FNA) are used to triage breast cancer patients to appropriate axillary surgery. We present a complete audit cycle. Departmental guidelines were changed to include repeat biopsy in cases with suspicious ultrasound/negative FNA and for patients with inconclusive FNA results between audit periods one and two.

## Methods

Retrospective analysis of multidisciplinary meeting and pathology records of all breast cancer patients operated upon in our Trust between January and June 2010, and then between January and December 2012, was reviewed. Descriptive statistics were performed.

## Results

Initial audit findings included 125 female patients, with overall combined sensitivity and specificity of ultrasound/FNA being 64% (21/33) and 100% (92/92) respectively. Included in the re-audit were 378 female patients, all underwent axillary ultrasound. Of these, 50.3% (190/378) patients underwent an FNA with 18.4% (35/190) having repeat FNA or core biopsy for inconclusive results. Of the repeat biopsies, 80% (28/35) were benign and 20% (7/35) were malignant. Ultrasound sensitivity was 78.9% (86/109). Sensitivity of FNA/biopsy was 82.6% (71/86). Overall combined sensitivity of ultrasound/FNA was 62.2% (74/119). Ultrasound specificity was 62.1% (167/269). Specificity of FNA was 97.1% (101/104). Overall combined specificity of ultrasound/FNA was 98.9% (266/269).

## Conclusion

Performing a repeat FNA/biopsy on inconclusive cases proved valuable in that 80% patients were spared from surgical morbidity. However, overall sensitivity of preoperative axillary staging has not significantly changed between cycles following resampling. Further audit, analysis and guideline review is needed to ensure optimal patient care.

